# Does continuous trusted adult support in childhood impart life-course resilience against adverse childhood experiences - a retrospective study on adult health-harming behaviours and mental well-being

**DOI:** 10.1186/s12888-017-1260-z

**Published:** 2017-03-23

**Authors:** Mark A. Bellis, Katie Hardcastle, Kat Ford, Karen Hughes, Kathryn Ashton, Zara Quigg, Nadia Butler

**Affiliations:** 1 College of Health and Behavioural Sciences, Normal Site, Bangor University, Bangor, LL57 2PZ UK; 2 Directorate of Policy, Research and International Development, Public Health Wales, Number 2 Capital Quarter, Tyndall Street, Cardiff, CF10 4BZ UK; 3 Public Health Institute, Faculty of Education, Health and Community, Liverpool John Moores University, 15-21 Webster Street, Liverpool, L3 2ET UK

**Keywords:** Resilience, ACEs, Non-communicable disease, Mental well-being, Deprivation, Parenting, Smoking, Alcohol, Diet

## Abstract

**Background:**

Adverse childhood experiences (ACEs) including child abuse and household problems (e.g. domestic violence) increase risks of poor health and mental well-being in adulthood. Factors such as having access to a trusted adult as a child may impart resilience against developing such negative outcomes. How much childhood adversity is mitigated by such resilience is poorly quantified. Here we test if access to a trusted adult in childhood is associated with reduced impacts of ACEs on adoption of health-harming behaviours and lower mental well-being in adults.

**Methods:**

Cross-sectional, face-to-face household surveys (aged 18–69 years, February-September 2015) examining ACEs suffered, always available adult (AAA) support from someone you trust in childhood and current diet, smoking, alcohol consumption and mental well-being were undertaken in four UK regions. Sampling used stratified random probability methods (*n* = 7,047). Analyses used chi squared, binary and multinomial logistic regression.

**Results:**

Adult prevalence of poor diet, daily smoking and heavier alcohol consumption increased with ACE count and decreased with AAA support in childhood. Prevalence of having any two such behaviours increased from 1.8% (0 ACEs, AAA support, most affluent quintile of residence) to 21.5% (≥4 ACEs, lacking AAA support, most deprived quintile). However, the increase was reduced to 7.1% with AAA support (≥4 ACEs, most deprived quintile). Lower mental well-being was 3.27 (95% CIs, 2.16–4.96) times more likely with ≥4 ACEs and AAA support from someone you trust in childhood (vs. 0 ACE, with AAA support) increasing to 8.32 (95% CIs, 6.53–10.61) times more likely with ≥4 ACEs but without AAA support in childhood. Multiple health-harming behaviours combined with lower mental well-being rose dramatically with ACE count and lack of AAA support in childhood (adjusted odds ratio 32.01, 95% CIs 18.31–55.98, ≥4 ACEs, without AAA support vs. 0 ACEs, with AAA support).

**Conclusions:**

Adverse childhood experiences negatively impact mental and physical health across the life-course. Such impacts may be substantively mitigated by always having support from an adult you trust in childhood. Developing resilience in children as well as reducing childhood adversity are critical if low mental well-being, health-harming behaviours and their combined contribution to non-communicable disease are to be reduced.

**Electronic supplementary material:**

The online version of this article (doi:10.1186/s12888-017-1260-z) contains supplementary material, which is available to authorized users.

## Background

An increasing international literature describes strong relationships between exposure to adverse childhood experiences (ACEs) and their impact on health across the life-course [1–4]. ACEs include being a victim of abuse or neglect as well as growing up in households in which there are issues such as domestic violence or adult substance use problems, long-term mental health conditions or criminal behaviour leading to incarceration of family members [1]. Exposure to ACEs is strongly associated with adopting health-harming behaviours (HHBs) in adolescence and adulthood such as smoking, heavier alcohol consumption, drug use and high calorie, low nutrient diets [1, 5–7]. Recent studies have shown that ACEs can alter early brain development including the pleasure and reward centres and can compromise the role of the pre-frontal cortex in impulse control [8, 9]. These and other changes result in lower tolerance for stress and consequently a greater propensity for anti-social behaviour (including violence) and difficulties feeling close to other people [10, 11]. Neurological changes related to chronic childhood stressors can also adversely impact cognitive functions affecting learning, memory and school performance [12]. Further, ACEs can impact the hypothalamic-pituitary-adrenal axis function, altering cortisol control and other hormonal and immunological systems, resulting in chronic tissue inflammation and increased allostatic load [13]. Such changes promote the earlier development of cancer, heart disease, diabetes and premature mortality [14–17].

A substantive subset of individuals who suffer ACEs avoid in part or entirely the negative health and social outcomes associated with chronically stressful childhoods; a characteristic referred to as resilience [18]. Resilience reflects an individual’s ability to transform potentially toxic stress into tolerable stress and consequently reduce the harmful physiological and psychological impacts of such stressors occurring during childhood development [19]. Emerging intelligence suggests a range of factors can help individuals develop resilience during childhood. Strong links with cultural traditions, better developed self-regulation skills and a sense of having some control over personal circumstances have all been associated with moderating the negative impacts of childhood adversity [19, 20]. Thus, when faced with a traumatic situation proximity to a caregiver is a critical contributor to a child’s sense of safety [21–23]. Such a history of being able to manage stressful situations may also lead to better adaptation to coping with stress as an adult [24, 25]. While many of the mechanisms underpinning resilience still require study, it appears to be an asset that can be developed prior to, during and after exposure to childhood adversity [19, 26].

In addition to ACEs and resilience-promoting factors, physical and mental health across the life-course can also be impacted by other factors, including deprivation [27, 28]. Economic gradients affect access to assets such as healthy diets and living environments as well as educational and employment opportunities. Moreover, poor social circumstances at any age can result in low self-esteem, feelings of lack of control over home and work environments and consequently long-term stress [29]. Poor access to health enabling assets and psychosocial factors associated with deprivation may directly affect mental health, adoption of HHBs and consequently, reduce years of life in good health [30–32]. Moreover, a history of exposure to ACEs and lower levels of resilience to physical and mental ill health have also been associated with deprivation [5, 33]. While many studies have examined the individual impacts of ACEs, resilience or deprivation on mental and physical health few have explored the relative contributions made by each. Here, while controlling for socio-economic factors, we test specifically whether access to a trusted adult in childhood is associated with reductions in the impacts of ACEs on adoption of heavy alcohol consumption, smoking, poor diet and low mental well-being among adults.

## Methods

A national household survey of adults resident in Wales was undertaken between February and May 2015 and repeated in three English geographical areas (Hertfordshire, Luton, Northamptonshire) between June and September 2015. Data collection used established ACE questions from the Centers for Disease Control and Prevention short ACE tool [34]. In total, 11 questions measured childhood exposure to abuse and family dysfunction occurring to respondents before the age of 18 years. These were reduced to nine categories of ACE covering: physical, verbal and sexual abuse; parental separation; exposure to domestic violence and growing up in a household with mental illness, alcohol abuse, drug abuse or with an individual who had been incarcerated (Additional file 1: Web Table 1). As elsewhere, individuals were categorised into having experienced 0, 1, 2–3 or ≥4 ACE categories [2]. Three current health-harming behaviours were measured (daily smoking, poor diet and regular heavy drinking) and for the purposes of analysis respondents were dichotomised (yes/no) for each variable into current daily smokers, typically consumed ≤1 portion of fruit or vegetables per day and drink six or more standard drinks on one occasion at least weekly (here, weekly heavy drinking sessions, [35] Additional file 1: Web Table 1). A wide range of literature indicates that the detrimental impacts on health of having more than one health-harming behaviour are multiplicative rather than additive [36–38]. Consequently, an additional dependent variable (reporting two or more health-harming behaviours, ≥2 HHBs) was also analysed. Other demographics collected were age categories, sex and ethnicity which was dichotomised into white and other ethnicities for the purposes of analysis due to relatively small numbers in each individual non-white ethnic group.

Mental well-being (MWB) was measured using the Short Warwick-Edinburgh Mental Well-being Scale (SWEMWBS)[39]. This measures how often over the past two weeks individuals have been: feeling optimistic about the future; feeling useful; feeling relaxed; dealing with problems well; thinking clearly; feeling close to other people and; able to make up their own mind about things. Each component is scored from 1 (none of the time) to 5 (all of the time) and a total mental well-being score is calculated (potential range lowest 7 to highest 35). As elsewhere, [11] lower mental well-being (LMWB) was defined as more than one standard deviation (4.85) below the mean (27.14) and consequently set for the purposes of analysis at ≤22. Finally, as one aspect of resilience, trusted adult support was measured by the question ‘While you were growing up, before the age of 18, was there an adult in your life who you could trust and talk to about any personal problems’. For the purposes of analyses, responses were dichotomised into those who did or did not always have trusted adult support during childhood (Always Available Adult [AAA] support, yes, no; Additional file 1: Web Table 1).

Sampling was undertaken using the national postcode address file to select households of residence basis [40] and households were selected through random probability sampling stratified by each of the four regions and then by small area deprivation using LSOAs; geographic areas with a population mean of 1,500) [41]. Within each region LSOAs were categorised into quintiles of deprivation based on their ranking in the English Index of Multiple Deprivation 2011 (IMD) [42] and, for Wales, the Welsh Index of Multiple Deprivation 2014 (WIMD) [43] and individuals assigned the deprivation quintile of their LSOA. Both IMDs use a composite deprivation measure based on domains including; income, employment, health, education, access to services, community safety and physical environment [44]. However, Welsh and English IMDs are not directly comparable. Therefore, deprivation quintiles were calculated separately for England and Wales and region of residence included as a potential confounder in all multi-variate analyses.

For selected addresses in England, letters were delivered to houses that outlined the study methodology as well as when the researchers might visit and provided information about how to opt out of the study. In Wales, researchers presented potential participants with a letter of authority upon each visit to households in the selected areas. Trained researchers visited the selected households on all days of the week between the hours of 9 am and 8 pm. Potential participants were presented with a copy of the study information sheet that outlined its purpose and provided information on the voluntary, confidential and anonymous nature of the survey. Individuals were informed that they were able to decline participation and were free to withdraw at any point and that doing so would not affect any other aspect of their health treatment or other services. After requesting their informed consent to proceed, the questionnaire was delivered to those agreeing and meeting the study inclusion criteria (aged 18–69 years; cognitively able to participate in a face-to-face interview and resident in the selected LSOA) by CAPI (computer-assisted personal interviewing) and CASI (computer-assisted self-interviewing) for some of the sensitive questions. As well as English language, respondents could opt to be interviewed in French, Spanish, Polish, Hindi, Punjabi, Urdu, Gujarati, Bengali, Marathi, Pashto, Sindhi, Saraiki and Balochi, and in Welsh for those surveyed in Wales. No personal identifiable details were collected from individuals at any stage during either the recruitment process or interview. Ethical approval was obtained from Liverpool John Moores University (England) and Public Health Wales (Wales) and the studies adhered to the Declaration of Helsinki.

Based on ACE prevalence identified in other UK surveys [5] overall sample size was set at approximately 7,500. In total 28,349 households were visited during the study periods but 42.8% (*n* = 12,127) did not result in any contact with a resident (e.g. unoccupied). Of the occupied households 20.8% (*n* = 3,371) were ineligible (e.g. out of age range or where language could not be accommodated). A further 32.1% (*n* = 5,200) declined to take part in the research, and 47.2% (*n* = 7,651) completed a questionnaire. Thus, based on known occupied eligible households overall compliance was 59.5%. Here however, any individuals who did not complete all questions required for these analyses were also removed resulting in a final sample of *n* = 7,047. Data input was undertaken in Microsoft Excel and all statistical analyses in SPSS v22. Analyses used chi squared for initial bivariate examination of associations with HHBs and LMWB. Subsequent multi-variate modelling employed binary and multinomial logistic regression in order to examine the independent contributions of ACEs, AAA support status, current deprivation and other demographic variables to outcomes of interest.

**Fig. 1:**
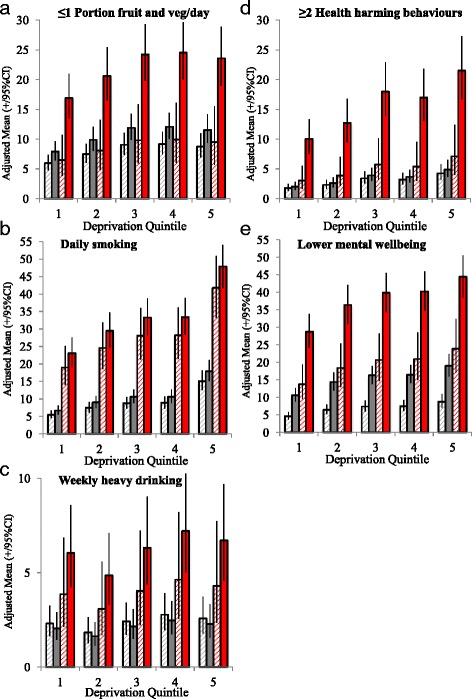
Adjusted means^$^ for mental well-being and health-harming behaviour outcomes by ACE count category and trusted adult support in childhood.  0 ACEs, Always available adult support in childhood = yes.  0 ACEs, Always available adult support in childhood = no.  ≥4 ACEs, Always available adult support in childhood = yes.  ≥4 ACEs, Always available adult support in childhood = no. Footnote. *ACE* Adverse Childhood Experiences. Graphical representations have been limited to ≥4 ACEs and 0 ACE categories for clarity of presentation. *95%CI* 95% Confidence Intervals. ^$^Adjusted means are calculated using estimated marginal means function and are adjusted through logistic regression modelling for confounding from other variables in the model; here age, sex, ethnicity (see Methods). Deprivation quintiles are from 1 = most affluent to 5 = most deprived.

To test for potentially different relationships by gender between dependent variables and ACEs with and without AAA support, an interactive term (gender by ACEs with and without AAA support) was included in all models but this did not contribute significantly in any instance. Modeled estimates for prevalence of dependent variables were calculated for different ACE counts, deprivation quintiles and trusted adult combinations using an estimated marginal mean function [45] to adjust all estimates to overall sample demographics (age, sex, ethnicity, region of residence). Adjusted means, with 95% confidence intervals, are shown for ACE counts (0 and ≥4 ACEs), with and without AAA support in childhood and across all deprivation quintiles (Fig. 1). Although risks of Type I errors were considered low, [46] key findings for ACEs and AAA support were also tested against adjusted significance levels using Bonferroni corrections.

## Results

In the final sample 18–29 years olds comprised the largest age group and 50–59 years olds the smallest; females represented 54.1%; and ethnicities other than white represented 15.2% (Table 1). Individuals resident in the most deprived quintile of deprivation comprised 12.9% compared to 26.7% in the most affluent (Table 1). Overall, 43.7% of individuals reported having experienced at least one ACE and 10.3% ≥4 ACEs. AAA status in childhood was reported by 46.4% of respondents (Table 1).

**Table 1:** Adverse childhood experiences, trusted adult support in childhood and demographic relationships with health-harming behaviours and mental well-being in adulthood

Category				Outcome (%)				
		n	%	Daily smoking	≤1 Portion fruit and veg/day	Weekly heavy drinking	≥2 Health-harming behaviours	Lower mental well-being
All		7047	-	19.1	12.6	7.7	8.5	15.5
ACE count	0	3964	56.3	14.1	10.5	5.7	5.6	11.5
	1	1271	18.0	18.3	13.5	8.3	8.5	14.2
	2–3	1086	15.4	22.3	13.0	9.4	9.8	18.1
	4+	726	10.3	43.4	22.3	15.6	22.9	35.4
	*X* ^*2*^			348.569	78.990	91.334	237.714	275.819
	P			<0.001	<0.001	<0.001	<0.001	<0.001
AAA support in childhood	Yes	3273	46.4	16.0	9.8	7.1	6.2	9.0
	No	3774	53.6	21.9	15.1	8.3	10.5	21.1
	*X* ^*2*^			39.187	45.067	3.914	41.286	196.572
	P			<0.001	<0.001	0.048	<0.001	<0.001
UK region	Luton	1334	18.9	18.1	10.9	4.0	6.7	14.5
	Wales	1819	25.8	22.3	20.2	12.7	14.7	19.4
	Hertfordshire	2421	34.4	17.7	10.0	7.1	6.9	13.8
	Northamptonshire	1473	20.9	18.5	9.2	5.9	5.3	14.4
	*X* ^*2*^			16.035	128.410	96.317	122.030	28.739
	P			0.001	<0.001	<0.001	<0.001	<0.001
Deprivation quintile	Affluent 1	1884	26.7	14.0	9.4	8.0	5.7	11.4
	2	1409	20.0	17.8	11.5	6.3	7.0	14.8
	3	1444	20.5	19.5	14.2	7.7	10.0	16.5
	4	1403	19.9	19.5	14.3	8.1	9.1	17.2
	Deprived 5	907	12.9	30.8	16.1	8.9	13.8	20.8
	*X* ^*2*^			112.981	35.957	6.230	60.510	49.141
	P			<0.001	<0.001	0.183	<0.001	<0.001
Age	18–29	1630	23.1	26.0	19.6	12.2	15.8	16.3
category	30–39	1423	20.2	19.7	9.1	6.6	6.8	15.7
(years)	40–49	1401	19.9	17.6	11.9	7.5	6.9	14.8
	50–59	1215	17.2	21.2	11.6	8.1	7.9	15.9
	60–69	1378	19.6	10.1	9.6	3.6	3.8	14.5
	*X* ^*2*^			128.350	101.589	82.286	160.650	2.394
	P			<0.001	<0.001	<0.001	<0.001	0.664
Sex	Female	3815	54.1	15.5	9.0	4.0	5.1	15.2
	Male	3232	45.9	23.4	16.9	12.1	12.6	15.8
	*X* ^*2*^			71.136	98.374	159.336	128.351	0.639
	P			<0.001	<0.001	<0.001	<0.001	0.424
Ethnicity	White	5976	84.8	20.9	13.0	8.9	9.4	16.0
	Other ethnicities	1071	15.2	9.4	10.8	1.5	3.9	12.5
	*X* ^*2*^			76.788	3.702	68.912	34.359	8.44
	P			<0.001	0.054	<0.001	<0.001	<0.001

*ACE* Adverse Childhood Experiences. *AAA* Always Available Adult support from someone trusted in childhood

### Individual health-harming behaviours (HHBs)

Bivariate analyses identified that the prevalence of each HHB increased with ACE count (Table 1). Always available adult (AAA) support in childhood was associated with lower levels of each HHB. There were also strong relationships between increasing deprivation and higher HHBs with the exception of alcohol where there was a more U shaped distribution (as reported elsewhere [47]). Area of residence, younger age and male gender were all significantly associated with each HHB. Ethnicity was not associated with poorer diet but white ethnicity was significantly related to higher levels of other HHBs. In order to account for any confounding affects of age, sex, ethnicity and other demographics a binary logistic regression model was employed. With a strong relationship between ACE count and adult support status (*X* ^2^ = 205.272, *P* <0.001; ACE count categories 0, 1, 2–3, ≥4; AAA support 50.9%, 50.0%, 41.3%, 23.4% respectively) these individual variables were included as a single variable categorised into all possible combinations of ACE category and adult support status (Table 2). Even among those with 0 ACEs, individuals without AAA support in childhood, had higher odds of smoking and poor diet. Increasing ACE counts without AAA support was strongly related to increases in odds of having each HHB (Table 2). With AAA support in childhood however, increasing ACE count had a limited impact on HHBs. Thus with AAA support, daily smoking was the only individual HHB that significantly increased at both 2–3 and ≥4 ACE counts (vs. 0 ACEs and AAA support). Modelled sample prevalence (see methods) by ACE count, AAA support in childhood and current deprivation reflect these results. Thus, ACE count in the poorest quintile increases poor diet prevalence in those without AAA support in childhood from 11.5% (0 ACEs) to 23.6% (≥4 ACEs). However, the increase with ACEs is eliminated when adult support was always present (Fig. 1a). For smoking however, while prevalence is again highest in individuals living in deprivation who have experienced ≥4 ACEs (47.9%), it remains at similar levels even with AAA support in childhood (41.8%, Fig. 1b). Consistent with studies elsewhere, HHBs were also highest in the youngest age groups, in males and in those of white ethnicity (Table 2) and may reflect higher baseline risk-taking in these groups [48]. Differences in particular in heavy drinking and poor diet were also apparent by region (Table 2) and may be related to cultural and related behavioural differences between the Welsh and English regions studied.

**Table 2:** Logistic regression analysis of adverse childhood experiences, trusted adult support in childhood, demographics and their associations with health-harming behaviours and mental well-being in adulthood

		Daily smoking 95% CIs				≤1 Portion fruit and veg/day 95% CIs				Weekly heavy drinking 95% CIs				≥2 Health- harming behaviours 95% CIs				Lower mental well-being 95% CIs			
		AOR	Low	High	P	AOR	Low	High	P	AOR	Low	High	P	AOR	Low	High	P	AOR	Low	High	P
^£^AAA support in childhood * ACEs																					
Yes	0	Ref			<0.001				<0.001				<0.001				<0.001				<0.001
	1	1.26	0.98	1.63	0.068	1.26	0.93	1.69	0.133	1.69	1.22	2.35	0.002	1.54	1.07	2.19	0.019	1.32	0.96	1.81	0.083
	2–3	1.65	1.26	2.17	<0.001	1.43	1.03	1.98	0.035	1.08	0.71	1.64	0.720	1.52	1.01	2.29	0.045	1.79	1.28	2.49	<0.001
	4+	4.04	2.84	5.74	<0.001	1.09	0.63	1.89	0.754	1.70	0.97	3.00	0.066	1.72	0.94	3.13	0.078	3.27	2.16	4.96	<0.001
No	0	1.23	1.02	1.48	0.032	1.36	1.10	1.68	0.004	0.89	0.67	1.17	0.392	1.15	0.87	1.53	0.325	2.44	1.98	3.01	<0.001
	1	1.58	1.23	2.01	<0.001	1.86	1.42	2.45	<0.001	0.94	0.64	1.38	0.748	1.67	1.17	2.39	0.005	2.95	2.27	3.83	<0.001
	2–3	1.96	1.55	2.47	<0.001	1.60	1.21	2.12	0.001	1.83	1.33	2.53	<0.001	2.28	1.63	3.17	<0.001	3.69	2.86	4.76	<0.001
	4+	5.16	4.14	6.44	<0.001	3.21	2.49	4.14	<0.001	2.72	2.01	3.69	<0.001	6.16	4.61	8.24	<0.001	8.32	6.53	10.61	<0.001
UK region	Luton Wales				0.109				<0.001				<0.001				<0.001				0.035
		0.80	0.65	0.98	0.030	1.86	1.47	2.35	<0.001	1.80	1.30	2.50	<0.001	1.50	1.13	1.99	0.005	1.28	1.03	1.59	0.026
	Hertfordshire	0.88	0.72	1.09	0.241	0.98	0.77	1.26	0.877	1.23	0.87	1.74	0.237	0.94	0.70	1.28	0.712	1.01	0.81	1.26	0.920
	Northamptonshire	0.80	0.64	0.99	0.040	0.89	0.68	1.17	0.412	0.91	0.62	1.31	0.602	0.62	0.44	0.87	0.005	1.07	0.84	1.35	0.600
Deprivation quintile	Affluent 1				<0.001				<0.001				0.093				<0.001				<0.001
	2	1.39	1.14	1.70	0.001	1.28	1.01	1.61	0.041	0.79	0.60	1.05	0.110	1.30	0.96	1.75	0.086	1.42	1.15	1.76	0.001
	3	1.66	1.37	2.02	<0.001	1.57	1.26	1.97	<0.001	1.05	0.80	1.37	0.732	1.96	1.48	2.58	<0.001	1.64	1.33	2.03	<0.001
	4	1.67	1.37	2.04	<0.001	1.60	1.27	2.01	<0.001	1.21	0.92	1.59	0.175	1.83	1.37	2.44	<0.001	1.67	1.35	2.07	<0.001
	Deprived 5	3.06	2.46	3.81	<0.001	1.52	1.17	1.96	0.002	1.12	0.82	1.53	0.490	2.45	1.80	3.33	<0.001	1.98	1.56	2.52	<0.001
Age	18–29				<0.001				<0.001				<0.001				<0.001				0.789
category	30–39	0.82	0.68	0.98	0.030	0.50	0.40	0.62	<0.001	0.65	0.50	0.86	0.002	0.49	0.38	0.63	<0.001	1.08	0.88	1.32	0.465
(years)	40–49	0.66	0.55	0.80	<0.001	0.64	0.52	0.79	<0.001	0.68	0.53	0.88	0.004	0.46	0.36	0.60	<0.001	0.97	0.79	1.19	0.748
	50–59	0.84	0.69	1.01	0.065	0.61	0.49	0.76	<0.001	0.70	0.53	0.91	0.007	0.53	0.40	0.68	<0.001	1.09	0.88	1.35	0.437
	60–69	0.34	0.28	0.43	<0.001	0.50	0.40	0.62	<0.001	0.29	0.21	0.40	<0.001	0.24	0.18	0.33	<0.001	1.01	0.82	1.25	0.912
Sex^$^	Male	1.79	1.58	2.04	<0.001	1.96	1.69	2.28	<0.001	3.39	2.78	4.13	<0.001	2.79	2.31	3.37	<0.001	1.01	0.88	1.15	0.923
Ethnicity^$^	Other	0.29	0.23	0.37	<0.001	0.88	0.70	1.12	0.307	0.16	0.10	0.27	<0.001	0.35	0.25	0.50	<0.001	0.77	0.62	0.96	0.019

*AAA* Always Available Adult support from someone trusted in childhood, *ACE* Adverse Childhood Experiences, *95%CIs* 95% Confidence Intervals, *AOR* Adjusted Odds Ratio, *Ref* Reference category and P values appearing in reference rows relate to testing of the overall contribution of each variable to the model. ^£^Combined interaction variable between trusted adult support in childhood and ACEs. ^$^For binary variables reference categories are female (for sex) and white (for ethnicity). Interactions between sex and the AAA support in childhood*ACEs variable were included in all models but did not reach significance in any of the models (daily smoking, *P* = 0.199; ≤1 portion of fruit and veg/day, *P* = 0.107; weekly heavy drinking, *P* = 0.472; ≥2 Health-harming behaviours, *P* = 0.211; lower mental well-being, *P* = 0.746)

### Multiple health-harming behaviours (≥2 *HHBs)*

Proportions of respondents with ≥2 HHBs increased strongly with ACE count from 5.6% of those with no ACEs to 22.9% of those with four or more but decreased with AAA support in childhood (Table 1). Increasing deprivation, male gender, younger ages and white ethnicity were also associated with higher prevalence of ≥2 HHBs (Table 1). These relationships all remained significant with logistic regression analysis. Odds of having ≥2 HHBs rose with increasing ACE count even with AAA support in childhood. However, the combined impact of lacking AAA support and increasing ACEs was substantially greater (Table 2). Thus, modelled sample prevalence rises from 7.1% in those resident in the most deprived quintile with ≥4 ACEs with AAA support in childhood to 21.5% in those without (Fig. 1d).

### Lower mental well-being (LMWB)

In bivariate analyses LMWB more than tripled with ACE count (0 vs. ≥4 ACEs, Table 1) and more than doubled when adult support was not always available in childhood (9.0–21.1%). LMWB was also associated with deprivation and white ethnicity in both bivariate and multivariate analyses (Tables 1&2). Counts of 2-3 or ≥4 ACEs significantly increased the odds of LMWB even with AAA support (≥4 ACEs vs. 0 ACEs, AAA support, AOR = 3.27, 95% CIs 2.16-4.96; Table 2). However, the combination of high ACE counts and lacking AAA support in childhood resulted in the highest increases in odds of LMWB (≥4 ACEs, without AAA support vs. 0 ACEs, with AAA support; AOR = 8.32, 95% CIs 6.53–10.61; Table 2). Thus, LMWB modelled sample prevalence increased from 23.9% in residents of the most deprived quintile with ≥4 ACEs and AAA support in childhood to 44.4% in those in the same quintile with ≥4 ACE but without such support (Fig. 1e).

### Health-harming behaviours with lower mental well-being

Finally, HHBs (e.g. smoking) can become particularly entrenched when combined with LMWB [49]. Thus, we used multinomial analysis to examine independent predictors of having ≥2 HHBs with LMWB. Having ≤1 HHB without LMWB was set as the reference category for all other potential outcomes (Table 3). Having ≥2 HHBs without LMWB was only significantly related to ACE count when individuals did not report AAA support in childhood (Table 3). However, odds of having ≥2 HHBs with LMWB (vs. ≤1HHB without LMWB) increased steeply with ACE count combined with a lack of AAA support as a child. Thus, in those with such support, having ≥2 HHBs with LMWB was 4.71 (95% CIs 1.68–13.23) times more likely in those with ≥4 ACEs (vs. 0 ACEs). However, the equivalent increase in odds for ≥4 ACEs and lacking AAA support in childhood was 32.01 (95% CIs 18.31–55.98; Table 3).

**Table 3:** Multinomial regression analysis of impact of adversity, trusted adult support in childhood and deprivation on relationships between health-harming behaviours and mental well-being in adulthood

			≥2 Health-harming behaviours good mental well-being 95% CIs				≤1 Health-harming behaviours lower mental well-being 95% CIs				≥2 Health-harming behaviours lower mental well-being 95% CIs			
		Ref Cat^£^	AOR	Low	High	P	AOR	Low	High	P	AOR	Low	High	P
Support in childhood ^£^AAA*ACES														
Yes	0	<0.001	1.40	0.94	2.09	0.098	1.21	0.86	1.72	0.273	2.36	1.12	4.97	0.024
	2–3		1.18	0.72	1.92	0.519	1.55	1.07	2.25	0.020	3.73	1.76	7.90	<0.001
	4+		1.47	0.71	3.05	0.297	3.17	2.03	4.94	<0.001	4.71	1.68	13.23	0.003
No	0		1.10	0.80	1.51	0.542	2.50	2.00	3.12	<0.001	2.18	1.19	3.99	0.012
	1		1.62	1.08	2.44	0.021	2.99	2.26	3.95	<0.001	3.46	1.71	7.02	<0.001
	2–3		1.87	1.25	2.79	0.002	3.46	2.63	4.56	<0.001	6.91	3.70	12.94	<0.001
	4+		4.47	3.11	6.43	<0.001	7.02	5.33	9.25	<0.001	32.01	18.31	55.98	<0.001
UK region	Luton	<0.001												
	Wales		1.54	1.09	2.16	0.013	1.27	1.00	1.61	0.054	1.60	1.01	2.53	0.046
	Hertfordshire		1.02	0.71	1.47	0.919	1.06	0.83	1.34	0.662	0.84	0.51	1.38	0.481
	Northamptonshire		0.63	0.42	0.96	0.030	1.13	0.88	1.46	0.332	0.63	0.36	1.10	0.103
Deprivation quintile	Affluent 1	<0.001												
	2		1.26	0.88	1.79	0.202	1.41	1.12	1.77	0.003	1.64	0.98	2.75	0.058
	3		1.63	1.16	2.28	0.005	1.47	1.17	1.85	<0.001	3.36	2.12	5.33	<0.001
	4		1.82	1.29	2.56	<0.001	1.65	1.31	2.08	<0.001	2.45	1.50	4.00	<0.001
	Deprived 5		2.73	1.90	3.91	<0.001	2.07	1.60	2.68	<0.001	3.02	1.79	5.07	<0.001
Age	18–29	<0.001												
category	30–39		0.37	0.27	0.52	<0.001	1.03	0.82	1.29	0.817	0.81	0.54	1.21	0.301
(years)	40–49		0.39	0.29	0.54	<0.001	0.95	0.76	1.20	0.673	0.64	0.42	0.97	0.036
	50–59		0.51	0.38	0.69	<0.001	1.13	0.90	1.43	0.301	0.59	0.38	0.94	0.027
	60–69		0.19	0.13	0.28	<0.001	1.03	0.82	1.30	0.801	0.40	0.24	0.66	<0.001
Sex^$^	Male	<0.001	2.68	2.13	3.36	<0.001	0.15	0.90	0.77	1.041	2.83	2.08	3.85	<0.001
Ethnicity^$^	Other	<0.001	0.34	0.22	0.52	<0.001	0.08	0.81	0.64	1.022	0.33	0.18	0.60	<0.001

*ACE* Adverse Childhood Experiences. ^*£*^ *Ref Cat* Reference category for dependent variable was ≤1 Health-harming behaviours and good mental well-being. *P* values in the Ref Cat column refer to the overall contribution on that variable to the model. *95% CIs* 95% Confidence Intervals, *AOR* Adjusted Odds Ratio. ^$^For binary variables reference categories are female (for sex) and white (for ethnicity). ^$^AAA, Always Available Adult support from someone trusted in childhood. AAA*ACE is a combined interaction variable between continuous trusted adult support and ACEs. An interaction between sex and the AAA support in childhood*ACEs variable was included in the model but did not reach significance (*P* = 0.394)

## Discussion

Consistent with other retrospective and prospective ACE studies [4–7, 11] results here identify strong relationships between exposure to ACEs as a child and adopting both HHBs and LMWB as an adult (Tables 1 & 2). However, risks appear to be mitigated substantively by having trusted adult support always available in childhood. While such support reduced risks of daily smoking, poor diet, ≥2 HHBs and LMWB risks were also exacerbated by deprivation even after ACE count had been taken into account (Tables 1&2). The relative impact of each factor varied with outcome examined (Fig. 1a-e). For example, higher ACE counts and deprivation both increased risk of reporting ≥2 HHBs but these were mitigated substantially by AAA support during childhood (Fig. 1d). Thus, for individuals reporting ≥4 ACEs and such support in childhood the adjusted prevalence of ≥2 HHBs more than doubled with deprivation from 3.0% (most affluent) to 7.1% (most deprived quintile). However, for those reporting ≥4 ACEs but lacking AAA support in childhood the respective adjusted prevalences of ≥2 HHBs were 10.1% (most affluent) and 21.5% (most deprived).

LMWB increased in prevalence with deprivation. However, across all deprivation quintiles, LMWB prevalence was almost halved in those with ≥4 ACEs and AAA support in childhood compared to those with ≥4 ACEs and no such support (Fig. 1e). A similar moderating impact of AAA support for those with ≥4 ACEs was also apparent with poor diet (Fig. 1a). Whilst AAA support in childhood also impacted risks of daily smoking the mitigating effects were smaller (Table 2) with ≥4 ACEs substantively increasing prevalence of smoking regardless of AAA support (Fig. 1b, Table 2). Examining the reasons for a smaller impact of trusted adults on smoking are beyond this study. However, the highly addictive qualities of tobacco, difficulties quitting especially when introduced to smoking early in life, peer pressure and persistent advertising in previous decades may all impact on any mitigating influences [50–52]. Consistent with other studies heavy drinking did not vary significantly with deprivation [38]. However, it was modified by the interactions between ACEs and trusted adult support in childhood (Table 2) resulting in higher levels of heavy drinking in those with ≥4 ACEs especially in the absence of AAA support during childhood (Fig. 1c). Overall however, these findings add to others suggesting that continuous trusted adult support as a child is one factor that promotes resilience and consequently can substantially mitigate the impacts of childhood adversity on life course behaviour and health [19, 22, 23].

LMWB is not only associated with higher uptake of HHBs but is also linked with lower likelihoods of reducing such behaviour [53] and consequently increased longer-term risks of non-communicable diseases (NCDs) [54]. Moreover, where more than one HHB co-occurs, individuals are at a multiplicative risk of developing diseases including liver disease, cancers and hypertension [36, 37, 55]. Results here suggest that high ACE counts are strongly linked with increased likelihoods of reporting LMWB combined with multiple HHBs. Thus, experiencing ≥4 ACEs without AAA support in childhood increased the odds of LMWB with ≥2 HHBs by over 30 times (vs. 0 ACEs with AAA support; Table 3). Consequently, preventing ACEs may be critical for reducing risks of some of the most damaging combinations of HHBs rooted in LMWB. Building resilience in children through developing supportive bonds with adults may substantively mitigate but not eradicate some of this additional risk (Table 3).

The importance of preventing ACEs including child abuse, neglect and exposure to domestic violence for both the well-being of children and their health trajectories across the life-course has attracted international attention. Two of the United Nations Sustainable Development Goals (SDGs, Gender Equity and, Peace, Justice and Strong Institutions [56]) and a recent World Health Resolution [57] for instance, focus on addressing violence against children and women. Early years parental support and pre-school enrichment programmes have been shown to improve child-parent relations and reduce child maltreatment [58]. Equally, other initiatives including paediatric screening for child abuse, maternal depression, domestic violence and parental substance use have also reported positive ACE prevention outcomes (e.g. Safe Environment for Every Kid) [59]. Eradication of ACEs remains a long-term aspiration and consequently, developing resilience in order to mitigate the impact of ACEs on health throughout the life course is a critical factor. Results here suggest that continuous trusted adult support may reduce the risks of multiple HHBs and LMWB by more than half (Fig. 1d, e). Such findings add to those describing how higher measures of resilience can counteract the negative impacts of ACEs on, for instance, educational outcomes [19]. Early parent-child support programmes that foster supportive adult-child relationships can help develop resilience as well as prevent ACEs. Further, interventions that build self-control and adaptive skills and help connect individuals with cultural traditions also strengthen resilience [60–62]. However, a relationship with a trusted adult has been suggested as the strongest component in resilience development [19]. Such relationships have been described as converting toxic stress from ACEs into tolerable stress by providing both mechanism and opportunity for stress response systems to return to their normal baselines. This protects brain and other body systems development from disruption while supporting growth in the coping skills of the child [19].

There are a number of important limitations relating to this study. Compliance was 59.3%. While this is comparable to other similar ACE studies we are unable to quantify any bias introduced by self-selection to participate. The study design was retrospective and therefore causality between outcomes and ACEs, deprivation and resilient factors cannot be established. Not all individuals were alcohol consumers and including those who did not drink alcohol for religious reasons could have impacted results. However, ethnicity was included as a factor in all multivariate analyses and repeating logistic regressions but excluding lifetime abstainers (results not shown) did not materially alter any relationships. Competition between topics for time and space in the ACE surveys meant we could only include a single question on trusted adult status and consequently we only measured one aspect of resilience. This measure was chosen based on previous reviews that have identified a trusted adult as a critical factor in resilience development [19, 22, 23]. However, further empirical work should explore which different adult roles (e.g. parental, other mentor) and features of trusted support (e.g. emotional, provision of safety and security) best foster resilience as well as how other social, educational and cultural factors can also promote resilience [63]. The experience of both ACEs and access to a trusted adult may also differ with individual factors such as gender. Our models identified no significant differences by gender in the relationships between exposure to ACEs with and without AAA status and either HHBs or LMWB (Tables 2 & 3). However, more detailed studies of such factors are required.

AAA status in childhood was also associated with higher ACE counts. We used multivariate techniques to identify the independent affects of ACEs and AAA status on HHB and LMWB (Tables 2, 3). However, more qualitative and longitudinal quantitative work is required to fully understand how these factors interact through potentially differing childhood histories and their consequent impact on health across the life course. Finally, we tested our key predictive variable (ACE count with and without AAA support) against five different individual or combined dependent variables (Table 2) with, in each case, the overall contribution of the variable being highly significant at *P* <0.00001. Corrections for multiple models (Bonferroni correction) did not affect the significant status of these results with P values remaining <0.001. Further, within each model, odds of each HHB and LMWB increased ordinally with increasing ACE counts (Table 2), indicative of non-random increases in odds and accompanying significance, rather than any randomly generated Type I error.

This research provides an initial examination of the interactions between a key factor in the development of resilience (AAA support in childhood), exposure to ACEs and their impact on HHBs and LMWB. More detailed studies are needed to address how other influences (e.g. cultural connectedness) contribute to resilience and how differences in sex, other demographics and length of exposure to both ACEs and resilience promoting factors in childhood alter their combined impact on HHBs and LMWB. Moreover, although not the focus of this study, AAA status in childhood showed small but significant variations with demography. Thus, AAA status was more frequently reported by females (49.4%, males 42.4%; *X* ^2^ = 40.104, *P* <0.001), in Northamptonshire (57.2% cv. Luton 42.8%, Wales 43.7%; Hertfordshire, 44.0%; *X* ^2^ = 86.512, *P* <0.001) and in those with white (47.3%) ethnicity (vs. BEM, 41.9%, *X* ^2^ = 10.382, *P* <.01). The underlying reasons for such variations may relate to cultural differences or other factors impacting endorsement of AAA status in childhood. However, further studies exploring perceived and real differences between groups in AAA status could help inform actions to increase resilience.

## Conclusions

Individuals exposed to ACEs develop poorer executive control over impulses, lower tolerance for stress and difficulties with trust and socialising [9–11]. Thus, such individuals appear physiologically predisposed to uptake of HHBs and development of LMWB; often with the former (alcohol, tobacco and high calorie eating) functioning as short-term coping mechanisms for the latter [1, 13, 64]. Our results support such relationships for each individual HHB. However, they also suggest the impact of ACEs is related to the adoption of multiple HHBs by the same individuals. Such behavioural combinations are disproportionately related to development of NCDs. While there have been extensive claims that resilience can mitigate such impacts of ACEs relatively little empirical work has been undertaken to examine the extent to which this occurs. Results here suggest having continuous access to a trusted adult in childhood may dramatically reduce the impacts of childhood adversity on mental well-being and adoption of HHBs and that these relationships are apparent across all socio-economic strata (Table 2, Fig. 1a-e). HHBs are a major cause of NCDs; the single largest cause of death in both high and low-middle income countries [65]. There is overwhelming evidence that poorly regulated pricing and advertising of alcohol, tobacco and high calorie-low nutrient foods pulls individuals into behaviours with high risks of NCDs [66, 67]. However, exposure to ACEs and low resilience development in early years push individuals towards the same harmful behaviours and must also be tackled if NCDs are to be reduced.

## Additional file

Additional file 1: Web Table 1. Adverse childhood experiences, health-harming behaviours and trusted adult status questions used in the survey. Description of data: Provides the questions used to measure the main outcomes used in the study and the qualifying responses. (DOCX 98 kb)

## Abbreviations

AAA: Always available adult support in childhood
ACE: Adverse childhood experience
AOR: Adjusted odds ratio
CI: Confidence interval
HHB: Health-harming behaviour
IMD: Index of multiple deprivation
LMWB: Lower mental well-being
LSOA: Lower super output area
MWB: Mental well-being
NCDs: Non-communicable diseases
WIMD: Welsh index of multiple deprivation
