# Erratum to: Does continuous trusted adult support in childhood impart life-course resilience against adverse childhood experiences - a retrospective study on adult health-harming behaviours and mental well-being

**DOI:** 10.1186/s12888-017-1305-3

**Published:** 2017-04-13

**Authors:** Mark A. Bellis, Katie Hardcastle, Kat Ford, Karen Hughes, Kathryn Ashton, Zara Quigg, Nadia Butler

**Affiliations:** 1 College of Health and Behavioural Sciences, Bangor University, Bangor, LL57 2PZ UK; 2 Directorate of Policy, Research and International Development, Public Health Wales, Number 2 Capital Quarter, Tyndall Street, Cardiff, CF10 4BZ UK; 3 Public Health Institute, Liverpool John Moores University, 5-21, Webster Street, Liverpool, L3 2ET UK

## Erratum

This article [1] has been updated to correct the legend to Fig. 1. The correct figure with the correct legend is also shown at the end of this erratum.

**Fig. 1:**
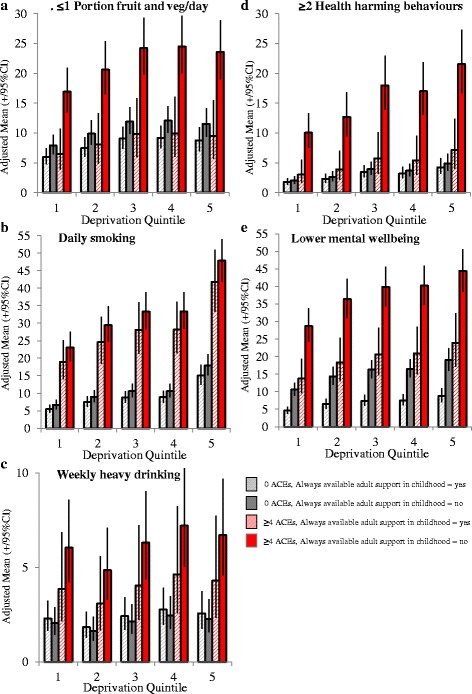
Adjusted means^$^ for mental well-being and health harming behaviour outcomes by ACE count category and trusted adult support in childhood. ACE=Adverse Childhood Experiences. Graphical representations have been limited to ≥4 ACEs and 0 ACE categories for clarity of presentation. 95% CI = 95% Confidence Intervals. ^$^Adjusted means are calculated using estimated marginal means function and are adjusted through logistic regression modelling for confounding from other variables in the model; here age, sex, ethnicity (see Methods). Deprivation quintiles are from 1 = most affluent to 5 = most deprived
