# Supplementary material for: Does continuous trusted adult support in childhood impart life-course resilience against adverse childhood experiences - a retrospective study on adult health-harming behaviours and mental well-being
Source: BMC Psychiatry. 2017 Mar 23;17:110. doi: 10.1186/s12888-017-1260-z (PMC5364707; doi:10.1186/s12888-017-1260-z)
Supplement: Additional file 1: Web Table 1. — Adverse childhood experiences, health-harming behaviours and trusted adult status questions used in the survey. Description of data: Provides the questions used to measure the main outcomes used in the study and the qualifying responses. (DOCX 98 kb) [file 12888_2017_1260_MOESM1_ESM.docx]

Additional file 1: Web Table 1. Adverse childhood experiences, health-harming behaviours and trusted adult status questions used in the survey^£^

| **ACE questions** were preceded by the statement “While you were growing up, before the age of 18...” | | | | |
| --- | --- | --- | --- | --- |
| **ACE** |  | **Question** |  | **Qualifying response^^^** |
| *Physical abuse* |  | How often did a parent or adult in your home ever hit, beat, kick, or physically hurt you in any way? This does not include gentle smacking for punishment. |  | Once or more than once |
| *Verbal abuse* |  | How often did a parent or adult in your home ever swear at you, insult you, or put you down? |  | More than once |
| *Sexual abuse* |  | How often did anyone at least 5 years older than you (including adults) ever touch you sexually? |  | Once or more than once to any of the questions |
| *Sexual abuse* |  | How often did anyone at least 5 years older than you (including adults) try to make you touch them sexually? |  |  |
| *Sexual abuse* |  | How often did anyone at least 5 years older than you (including adults) force you to have any type of sexual intercourse (oral, anal, or vaginal)? |  |  |
| *Parental separation* |  | Were your parents ever separated or divorced? |  | Yes |
| *Domestic violence* |  | How often did your parents or adults in your home ever slap, hit, kick, punch, or beat each other up? |  | Once or more than once |
| *Mental illness* |  | Did you live with anyone who was depressed, mentally ill, or suicidal? |  | Yes |
| *Alcohol abuse* |  | Did you live with anyone who was a problem drinker or alcoholic? |  | Yes |
| *Drug abuse* |  | Did you live with anyone who used illegal street drugs or who abused prescription medications? |  | Yes |
| *Incarceration* |  | Did you live with anyone who served time or was sentenced to serve time in a prison or young offenders' institution? |  | Yes |
| **Health-harming behaviours** |  |  |  |  |
| *Poor diet* |  | On a normal day, how many portions of fruit and vegetables (excluding potatoes) would you usually eat (one portion is roughly one handful or a full piece of fruit such as an apple)? |  | ≤1 portion of fruit or vegetables per day |
| *Smoking* |  | In terms of smoking tobacco, which of the following best describes you? |  | Daily smoking |
| *Heavy alcohol consumption^$^* |  | How often do you have 6 or more standard drinks on one occasion |  | Weekly or more frequently |
| **Trusted adult status^i^** |  | While you were growing up, before the age of 18, was there an adult in your life who you could trust and talk to about any personal problems |  | Always^*^ |

^£^ The Short Warwick-Edinburgh Mental Well-Being Scale (SWEMWBS) was used to assess mental well-being. SWEMWBS questions are available in full elsewhere and therefore not listed here (see Methods). ^^^Responses listed are those categorised here as an ACE. ^$^Questions on alcohol consumption were drawn from the AUDIT-C tool and participants were provided with information on what constitutes a standard UK drink (UK = 8mg of alcohol). ^*^For the purposes of analyses, responses (never, sometimes, always) were dichotomised into those who did or did not always have trusted adult support available to them during childhood (Always Available Adult [AAA] support, yes, no).
